# The Primary Victims of War—A Commentary on: “*The Disproportionate Surgical Burden Borne by Children in Regions of Armed Conflict*”

**DOI:** 10.1002/wjs.12652

**Published:** 2025-06-04

**Authors:** Wafa Audei, Kokila Lakhoo

**Affiliations:** ^1^ Nuffield Department of Surgical Sciences University of Oxford Oxford UK; ^2^ Oxford Paediatric Surgery Unit Oxford University Hospitals NHS Foundation Trust Oxford UK

**Keywords:** children, conflict, Congo, Gaza, Sudan, surgery, Syria, Ukraine, war, Yemen

At a quarter of the way through the 21st century the world is witnessing multiple ongoing conflicts, rising geopolitical tensions, and the risk of further escalation. It is therefore crucial that the global surgical community advocates for what is arguably its most vulnerable patients, namely children in active war zones. The authors examine the unique challenges of paediatric surgery in modern warfare, focusing on six active conflict zones in the Democratic Republic of Congo (DRC), Syria, Yemen, Ukraine, Sudan, and Gaza, while also proposing strategies to enhance surgical outcomes [1].

As demonstrated by the presented data, wars tend to disproportionately impact low‐ and middle‐income countries (LMICs) with fragile health infrastructures. Coupled with a typically higher paediatric population in conflict regions and the growing prevalence of urban warfare, this leads to devastating consequences. The paper highlights that nearly 15 million children in the DRC and 14 million in Sudan require humanitarian aid compared to 1 million in Gaza and 4 million in Ukraine, underscoring the imbalance in global media coverage [1].

Children in war settings experience significantly higher mortality and morbidity rates than adults due to their physiological vulnerabilities and the complexity of their injuries. Managing paediatric trauma in conflict zones presents unique challenges, as differences in anatomy and physiology necessitate specialised surgical and anaesthetic care, which is often unavailable in LMICs. Limited medical resources further complicates care, raising ethical dilemmas in triage and prioritisation. Beyond immediate surgical needs, children in war endure long‐term consequences, including physical disabilities, psychological trauma, loss of family, and restricted access to rehabilitation. Addressing these challenges requires sustainable interventions through training and capacity‐building of local healthcare providers, thus ensuring continuity of care in conflict‐affected regions.

Children possess intrinsic moral value and warrant special ethical consideration, particularly in the context of war where they are innocent parties and their vulnerability is heightened. Bioethics underscores the principle that children should not be seen merely as passive recipients of care but as individuals with rights and intrinsic dignity [2]. Armed conflict exposes children to extreme physical and psychological harm, violating their fundamental rights to health, security, and well‐being. Given their unique developmental needs and future potential, the global community has an ethical duty to prioritise paediatric care in war zones, ensuring that interventions are guided not only by medical necessity, but also by a broader commitment to justice, protection, and equity.

Surgery has historically advanced through the harsh lessons of warfare, with the First and Second World Wars serving as pivotal points in the development of trauma and reconstructive surgery. The scale and complexity of injuries demanded novel techniques, including skin grafting and craniofacial reconstruction, laying the foundations for modern practice. These wartime innovations underscore the role of necessity‐driven research and systematic data collection. Documenting and analysing surgical care in conflict settings among vulnerable populations, such as children, can refine techniques and strengthen evidence‐based practices.

Children are disproportionately affected in modern conflicts, with a higher incidence of blast injuries. A systematic review by *Wild et al.* revealed that 57% of injuries among children in war zones are due to blasts, nearly double the 24.8% in adults. These often require complex interventions, including amputations, wound debridement, and fracture fixations. Despite injury severity, mortality rates among children were only slightly higher than those in adults (11.0% vs. 9.8%; *p* < 0.05). However, the burden is greater in terms of disability‐adjusted life years (DALYs) due to the long‐term impact of injury sustained earlier in life. The review also highlighted inconsistencies in data reporting, underscoring the need for standardised data collection to optimise care for paediatric casualties [3].

*Samad et al.* estimate that 1.7 billion children worldwide lack access to surgical care, particularly in LMICs where surgical workforce density is alarmingly low. Malawi and Pakistan report only 0.17 and 0.4 paediatric surgeons per million children respectively [4]. This shortage is worsened by conflict. Studies on the economic consequences of child casualties are scarce. A review by *Kadir et al.* notes that children account for a large portion of war‐related deaths and disappearances, with malnutrition and disease as leading causes. These outcomes impede the development of human capital, which is essential for economic growth [5].

Improving paediatric surgery in conflict zones requires a coordinated and multidisciplinary effort to drive meaningful change. As the authors have emphasised, standardised data collection is essential to understand the full scope of paediatric surgical needs in war zones. This will allow for evidence‐based, targeted interventions that can improve outcomes and inform global health responses. There is an urgent need for an international Delphi process to establish consensus on standardised data collection practices in war. Further research should also quantify the economic impact of child mortality and morbidity, particularly in DALYs, to better inform policy and resource allocation.

While frameworks such as the UN Security Council Resolution 2427 call for the integration of child protection into peace processes, implementation remains limited—with fewer than one in five peace agreements including child‐specific provisions [1]. This underscores the need for sustained political lobbying and advocacy from the global surgical and health communities. The international community has a legal and moral obligation to uphold international humanitarian law (IHL) by safeguarding healthcare workers and facilities, enabling the safe passage of medical aid through humanitarian corridors, and supporting the International Court of Justice in actively prosecuting those responsible for war crimes. These actions are essential to establishing a more equitable, resilient, and child‐focused framework for delivering surgical care in conflict‐affected regions.

In summary, the authors shed light on the grave and often overlooked burden borne by children requiring surgical care in conflict zones. Their unique vulnerabilities, compounded by fragile health systems and insufficient resources, demand urgent, coordinated action from the global health community and humanitarian organisations. From data collection to workforce development and political advocacy, a multifaceted and sustained effort is essential. Ultimately, safeguarding children in war is not a solitary endeavor—it takes a global village to protect and heal a child amidst the chaos of conflict.

## Author Contributions

**Wafa Audei:** conceptualization, writing – original draft. **Kokila Lakhoo:** writing – review and editing.

## Conflicts of Interest

The authors declare no conflicts of interest.
